# Abietic Acid Induces DNA Damage and Cell Apoptosis in Lung Cancer Cells Through Targeting TOP2A

**DOI:** 10.3390/biom15111498

**Published:** 2025-10-24

**Authors:** Zhiyu Zhu, Jie Gu, Zehua Liao, Mengting Chen, Yun Wang, Jingyi Song, Jing Xia, Xinbing Sui, Shuang Lin, Xueni Sun

**Affiliations:** 1 School of Pharmacy, Hangzhou Normal University, Hangzhou 311121, China; 2 College of Life and Environmental Sciences, Hangzhou Normal University, Hangzhou 311121, China; 3 Department of Thoracic Surgery, The First Affiliated Hospital, College of Medicine, Zhejiang University, Hangzhou 310058, China

**Keywords:** abietic acid, natural products, lung cancer, TOP2A, DNA damage

## Abstract

**Background**: This study investigated the therapeutic effects and underlying mechanisms of abietic acid, an abietane diterpene extracted from *Pimenta racemosa* var. *grissea*, against lung cancer. **Methods**: Initially, cell viability, colony formation, flow cytometry, and mitochondrial membrane potential detection were conducted to determine the impact of abietic acid on lung cancer cells. Subsequently, the antitumor mechanisms of abietic acid were predicted using network pharmacology and validated via immunofluorescence, reactive oxygen species (ROS) detection, molecular docking, gene knockdown techniques and Western blotting. Finally, an in vivo xenograft model assessed its tumor-suppressive potential, with Hematoxylin–Eosin (H&E) staining, Western blotting, and immunohistochemistry performed to examine pathological changes and protein expression alterations. **Results**: The proliferation of lung cancer cells was significantly inhibited by abietic acid. Additionally, abietic acid induced apoptosis and reduced mitochondrial membrane potential. Network pharmacology and Gene Ontology (GO) enrichment analysis revealed that the DNA damage response was a key biological process affected by abietic acid. Further results demonstrated that abietic acid induces DNA damage in lung cancer cells through targeting DNA topoisomerase II alpha (TOP2A). In vivo studies confirmed the antitumor efficacy of abietic acid and its low systemic toxicity. **Conclusions**: Abietic acid demonstrated significant antitumor effects in lung cancer cells by downregulating TOP2A, which induced DNA damage and apoptosis, revealing its clinical potential.

## 1. Introduction

Lung cancer is one of the most frequently occurring cancers worldwide and is the deadliest in terms of cancer-related fatalities [1]. According to global cancer statistics for 2022, lung cancer ranks first in incidence and mortality worldwide [2]. Current therapeutic approaches for lung cancer usually include surgical resection, radiotherapy, chemotherapy, immunotherapy and molecular targeted therapy, which are administered as monotherapy or multimodal interventions [3]. Despite these treatment strategies extending the survival of patients to some degree, the general outlook for lung cancer patients is still bleak, with only about 20% surviving for five years [4,5]. Therefore, it is crucial to develop more effective and safer therapeutic agents for lung cancer.

It is well known that traditional Chinese medicine (TCM) is a rich source of natural products [6,7,8,9,10]. In recent decades, natural products have been widely utilized in the treatment of various diseases, including cancer, and have been a primary source for the discovery of numerous active pharmaceutical ingredients [11]. Compared to synthetic compounds, natural products offer advantages such as structural diversity and complexity [12]. Natural products are characterized by mild effects, low toxicity, and multi-target activity, which makes them widely used not only for the prevention and management of early cancer symptoms but also in the treatment of advanced cancer [13]. Additionally, certain natural products and their derivatives have demonstrated superior efficacy in targeting drug-resistant cancer cells [14]. Consequently, the exploration of natural products for cancer therapy has gained increasing significance in the field of drug discovery and research.

Abietic acid (AA) is an abietane diterpene extracted from *Pimenta racemosa* var. *grissea*, known for its anti-allergic, anti-inflammatory, anti-obesity, and anticonvulsant activities [15,16,17,18]. Previous studies have demonstrated that abietic acid and its derivatives exhibit promising antitumor effects [19]. Specifically, abietic acid has been shown to inhibit metastasis and invasion in melanoma models both in vitro and in vivo [20], induce ferroptosis in bladder cancer cells by activating the heme oxygenase-1 (HO-1) pathway [21], induce apoptosis in breast cancer cells by regulating diverse gene expression patterns [22], and suppress the growth of non-small cell lung cancer (NSCLC) cells by inhibiting IκB kinase β (IKKβ)/nuclear factor-κB (NF-κB) signaling [23]. Despite these findings, research investigating the anticancer effects and underlying mechanisms of abietic acid in lung cancer remains in its early stages.

This study explored how abietic acid influences proliferation, apoptosis, and DNA damage in lung cancer cells. To investigate the molecular mechanisms responsible for the anticancer properties of abietic acid, network pharmacology was employed to identify key targets and signaling pathways. The identified target was subsequently verified through molecular docking and Western blotting analysis. Furthermore, the antitumor effect of abietic acid was evaluated in vivo. The findings reveal that abietic acid may be a potential therapeutic candidate for lung cancer by inducing DNA damage. This study establishes a scientific foundation for the suppressive effects of abietic acid on lung cancer and offers fresh perspectives on the application of TCM in lung cancer treatment.

## 2. Materials and Methods

### 2.1. Cell Culture and Treatment

The human lung cancer cell lines NCI-H460 (H460) and NCI-H1975 (H1975) and human embryonic lung fibroblast (HELF) cells were purchased by the Cell Bank of the Chinese Academy of Sciences (Shanghai, China). Cells were maintained in a full growth medium containing 10% FBS and 1% penicillin–streptomycin, and kept at 37 °C under a humidified atmosphere with 5% CO_2_. Cells were treated with abietic acid (Shanghai Yuanye Bio-Technology, B21212, Shanghai, China) at concentrations of 0, 200, 300, and 400 μM for either 24 or 48 h.

### 2.2. Cytotoxicity Testing

The cytotoxic effects of abietic acid were evaluated using the CCK-8 viability assay (MA0218-5, Meilunbio, Dalian, China). A total of 5 × 10^3^ cells/well were seeded in 96-well plates and treated with different concentrations of abietic acid for 24 or 48 h. Subsequently, the absorbances were detected at 450 nm using microplate readers (Thermo Scientific, Waltham, MA, USA) following the addition of CCK-8 to each well.

### 2.3. Colony-Formation Assay

A total of 5 × 10^3^ cells/well were plated in 10 cm dishes and treated with abietic acid for 48 h, followed by a further 10 days of culture. Following the processes of paraformaldehyde fixation and crystal violet staining, the cell clusters were visualized and quantified.

### 2.4. Apoptosis and ROS Assay

After cells were treated with abietic acid for 48 h, they were collected and stained with Annexin V-FITC/PI apoptosis detection kit (40302ES60, YEASEN, Shanghai, China) or ROS assay kit (S0033M, Beyotime, Shanghai, China). Both apoptosis and ROS levels were performed using flow cytometry (Beckman Coulter CytoFLEX, Brea, CA, USA). The data were processed using CytExpert 2.4 software.

### 2.5. Mitochondrial Membrane Potential Assay

Loss of mitochondrial membrane potential was evaluated using a commercial mitochondrial membrane potential assay kit (C2003S, Beyotime, Shanghai, China). After being stained with JC-1 dye at a concentration of 1× in staining buffer for 30 min, the cells underwent three washing cycles with buffer prior to being imaged using fluorescence microscopy (Thermo Scientific, Waltham, MA, USA).

### 2.6. Western Blotting

Total cellular proteins were isolated with RIPA buffer. Following BCA-based protein quantification, equal amounts of protein lysates were separated by SDS-PAGE and transferred to PVDF membranes, which were then blocked with 5% skim milk. After incubating the membranes with primary antibodies overnight at 4 °C, they were incubated with secondary antibodies at room temperature for 2 h. Protein bands were subsequently developed using enhanced chemiluminescence. The antibodies used were as follows: GAPDH (1:50,000; A19056), Bax (1:1000; A19684), anti-mouse IgG (1:2000; AS003) and Anti-Rabbit IgG (1:2000; AS014), all from ABclonal Technology (Woburn, MA, USA). γH2A.X (1:1000; 80312), cleaved PARP (1:1000; 5625), cleaved caspase-3 (1:1000; 9664) and Bcl-2 (1:1000; 15071) were obtained from Cell Signaling Technology (Danvers, MA, USA). TOP2A (1:1000; ET1607-59), p-Chk1 (1:1000; ET1611-76), p-Chk2 (1:2000; HA721633), p-ATM (1:1000; ET1705-50) and p-ATR (1:1000; HA721190) were provided by HUABIO (Woburn, MA, USA).

### 2.7. Target Exploration of Abietic Acid in Lung Cancer

CHEMBL (https://www.ebi.ac.uk/chembl/, accessed on 5 August 2023) is a comprehensive bioactivity database for drug-like molecules [24]. The CHEMBL database was employed to predict potential targets of abietic acid in humans, which were predicted by selecting the 100 highest-ranking targets according to an activity threshold. The conversion of CHEMBL IDs to gene names was facilitated by the Uniprot database (https://www.uniprot.org/, accessed on 5 August 2023). Additional potential targets of abietic acid were predicted using NetInfer (http://lmmd.ecust.edu.cn/netinfer/, accessed on 5 August 2023), a public online database for target prediction [25]. Targets that achieved a score above 1.0 in the NetInfer database were selected for analysis. SwissTargetPrediction (https://swisstargetprediction.ch/, accessed on 5 August 2023) is an online tool for predicting the most likely protein targets of small molecules [26]. This tool was also utilized to predict targets and those with a probability greater than 0.1 were selected. Disgenet (https://www.disgenet.org/, accessed on 5 August 2023) is a collaborative database of disease-related genes that can be utilized to make predictions concerning potential targets for lung cancer [27]. The Disgenet database was used to identify potential therapeutic targets, with a selection threshold of a score greater than 0.1. Finally, common targets for abietic acid against lung cancer were obtained by the jvenn online tool (https://jvenn.toulouse.inra.fr/app/index.html, accessed on 5 August 2023) [28].

### 2.8. PPI Network Analysis

Protein–protein interaction (PPI) analysis was conducted using the STRING database (https://string-db.org/, accessed on 14 August 2023). Common targets were analyzed in the STRING database (v12.0), with homo sapiens set as the organism and interaction confidence threshold set to 0.400. The PPI network data was imported into Cytoscape (v3.9.1) for visualization.

### 2.9. Bioinformatics Analysis and Enrichment Analysis

The GEPIA database (http://gepia.cancer-pku.cn/, accessed on 20 August 2024) was used to analysis the differential expression of the targets between normal and lung cancer tissues and the overall survival of patients with differential expression of the targets. Correlations between lung cancer patient survival and TOP2A expression were analyzed by Kaplan–Meier plotter (http://kmplot.com, accessed on 30 September 2025). Pearson correlation analysis was employed to pinpoint the genes most closely linked to TOP2A within the GSE268175 dataset in the GEO database (https://www.ncbi.nlm.nih.gov/geo/, accessed on 20 August 2024). Gene enrichment analysis was conducted by using the DAVID database (https://davidbioinformatics.nih.gov, accessed on 20 August 2024).

### 2.10. Immunofluorescence

The cells were seeded onto coverslips and treated with abietic acid for 48 h. Following fixation in paraformaldehyde and permeabilization with Triton X-100 at room temperature, the cells were blocked with BSA at 37 °C for 30 min. The cells were stained with γH2A.X (1:200; 80312, CST) overnight at 4 °C, then incubated with the fluorescent secondary antibody (1:500; 4408, CST) working solution at 37 °C for 1 h. The wavelength for fluorescent secondary antibody was 488 nm. Finally, the cells were counterstained with Hoechst and observed under a confocal microscope (Olympus, Tokyo, Japan).

### 2.11. Molecular Docking

Discovery Studio 2019 was used to conduct molecular docking with abietic acid and candidate targets. The chemical compounds and target proteins were obtained from PubChem and PDB database, respectively. Prior to the molecular docking analysis, the target protein was treated, which included the removal of water molecules, hydrogenation, and the identification of the active pocket. The docking analysis was evaluated using CDOCKER interaction energy, ChiFlex energy, and LibDockScore.

### 2.12. LDH Release Assay

After the indicated treatments, lactate dehydrogenase (LDH) release was determined using a LDH assay kit (C0016, Beyotime Biotechnology, Shanghai, China) according to the manufacturer’s protocol.

### 2.13. Real-Time Quantitative PCR (RT-qPCR)

Total cellular RNA isolated via RNAex Pro Reagent (AG21102, Accurate Biology, Changsha, China) was reversed-transcribed to cDNA using the HiScript III RT SuperMix for qPCR (Vazyme, R323, Nanjing, China). SYBR Mix (Vazyme, Q711) was used to analyze the mRNA expression. The primer sequences were as follows: TOP2A: 5′-3′ (Forward) TTCTTGATATGCCCCTTTGG, 5′-3′ (Reverse) GCTTCAACAGCCTCCAATTC; GAPDH: 5′-3′ (Forward) GGAGCGAGATCCCTCCAAAAT, 5′-3′ (Reverse) GGCTGTTGTCATACTTCTCATGG.

### 2.14. Knockdown of TOP2A

TOP2A knockdown in lung cancer cells was accomplished through transient transfection with siRNA-TOP2A using AccuFect RNAi transfection kit (AG51018, Accurate Biology). After 24 h of transfection, the cells were harvested for RT-qPCR analysis or treated with abietic acid for a further 48 h, after which Western blotting and CCK-8 assays were performed. Primer sequences for knockdown are listed as follows: siTOP2A-1: 5′-3′ (sense) GCUGCGGACAACAAACAAATT, 5′-3′ (antisense) UUUGUUUGUUGUCCGCAGCTT. siTOP2A-2: 5′-3′ (sense) GGGCAAAGAAACCUAUAAATT, 5′-3′ (antisense) UUUAUAGGUUUCUUUGCCCTT.

### 2.15. In Vivo Therapeutic Study

The animal experiment was approved by the animal ethics committee of Hangzhou Normal University (approval number: HSD-20250428-04). A total of 5 × 10^6^ H460 cells were inoculated subcutaneously into the nude mouse. The health of the experimental mice was monitored daily. The mice were randomly assigned to the control group, low-dose abietic acid group (200 mg/kg), high-dose abietic acid group (400 mg/kg). Tumor size was measured daily from day 0 to day 18 using a caliper. The body weight, length (L), and width (W) of the tumors were recorded, and tumor volume was determined using the following formula V = (L × W^2^)/2. Finally, the mice were sacrificed, all tumors and organ tissues were gathered, and blood biochemistry was analyzed for Alanine Transaminase (ALT), Aspartate Aminotransferase (AST), Creatinine (Cr) and Creatine Kinase (CK) by the Animal Center of Hangzhou Normal University.

### 2.16. H&E Staining

Tumor and organ tissue were preserved in 4% paraformaldehyde, embedded in paraffin, and sectioned into 5 μm slices. Sections were put on glass slides, stained with hematoxylin–eosin according to the kit instructions, and observed using a fully automated slide scanning imaging system (Olympus, Tokyo, Japan).

### 2.17. Immunohistochemistry (IHC) Analysis

After dewaxing and rehydrating the tumor sections, antigen retrieval was performed in a citrate buffer. Endogenous peroxidase activity was blocked, and then the sections were exposed to 3% H_2_O_2_ and 10% goat serum to inhibit endogenous peroxidase activity and non-specific protein binding. The sections were incubated with the primary antibodies overnight at 4 °C. The sections were then treated with polymer-conjugated secondary antibodies at 37 °C for 2 h, stained with DAB, and counterstained with hematoxylin. The antibodies used were as follows: γH2A.X (1:200; 80312), cleaved caspase-3 (1:2000; 9664) and ki67 (1:1000; 9449) were obtained from Cell Signaling Technology. TOP2A (1:1000; ET1607-59) were provided by HUABIO. TUNEL Apoptosis Detection Kit (DAB, Brightfield; HKI0012) was purchased from Haoke Biotechnology (Hangzhou, Zhejiang, China).

### 2.18. Statistical Analysis

Statistical analyses were conducted in GraphPad Prism 8.3. Statistical significance was assessed using ANOVA followed by Dunnett’s and/or Tukey’s multiple comparison test. Data represent mean ± SD from three replicates, with *p* < 0.05 considered statistically significant.

## 3. Results

### 3.1. Abietic Acid Inhibits the Proliferation of Lung Cancer Cells

**Figure 1:**
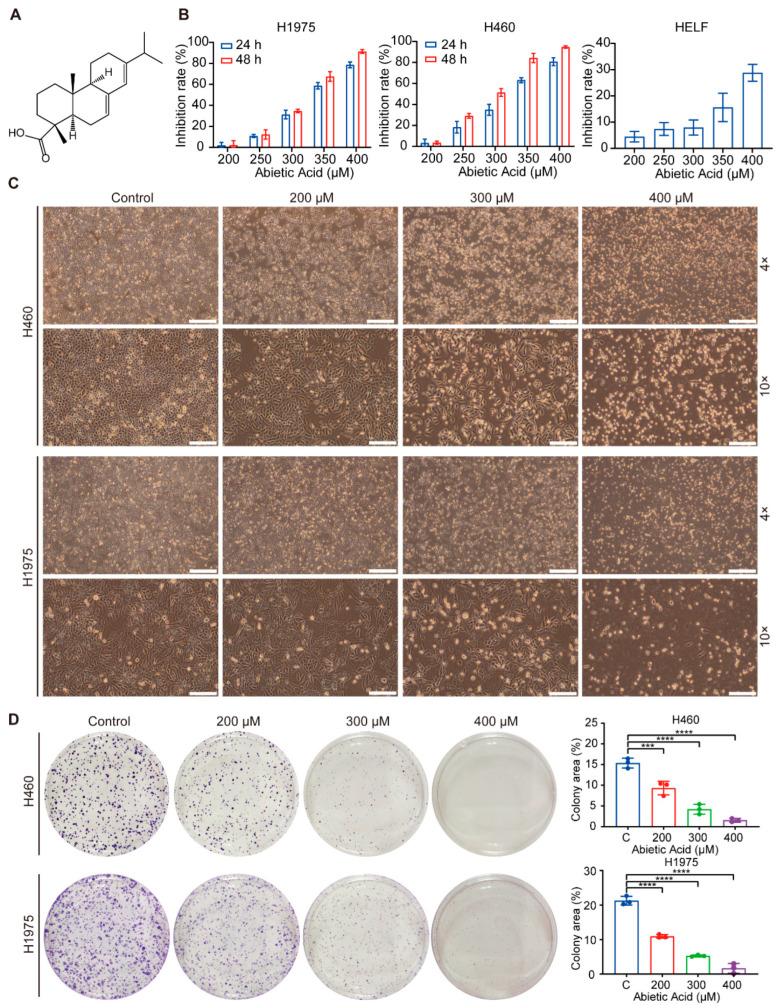
Abietic acid suppress the growth of lung cancer cells. (**A**) The molecular structure of abietic acid. (**B**) The cell viability of H460, H1975 and HELF cells was evaluated using a CCK-8 assay. (**C**) Changes in cell morphology were observed under a microscope after 48 h of abietic acid treatment. Scale bars correspond to 500 μm and 200 μm, respectively. (**D**) The colony formation assay results for H460 and H1975 cells after exposure to varying levels of abietic acid: mean ± SD; *n* = 3; *** *p* < 0.001; **** *p* < 0.0001.

Abietic acid, an abietane diterpene compound, is structurally illustrated in Figure 1A. To assess its cytotoxic effects, normal cell lines (HELF) and lung cancer cell lines (H460 and H1975) were exposed to different doses of abietic acid for 24 and 48 h. Cell viability was subsequently assessed using CCK-8. The results showed that cell proliferation was inhibited in a concentration-dependent manner in both lung cancer cell lines, whereas normal HELF cells exhibited minimal cytotoxicity (Figure 1B). The calculated IC_50_ values of abietic acid in H460 and H1975 cells were 323.2 μM and 334.0 μM at 24 h, and 290.8 μM and 320.2 μM at 48 h, respectively. Consistent with these results, microscopic observations revealed a substantial reduction in cell density and morphological changes indicative of suppressed proliferation in abietic acid-treated cancer cells (Figure 1C). To further evaluate the long-term proliferative potential of these cells, colony formation assays were subsequently performed. The results demonstrated that abietic acid substantially inhibited the colony-forming ability of both H460 and H1975 cells (Figure 1D). These findings collectively provide strong evidence that abietic acid exerts significant anti-proliferative effects against lung cancer cells while exhibiting low toxicity toward normal cells.

### 3.2. Abietic Acid Induces Mitochondrial-Related Apoptosis in Lung Cancer Cells

**Figure 2:**
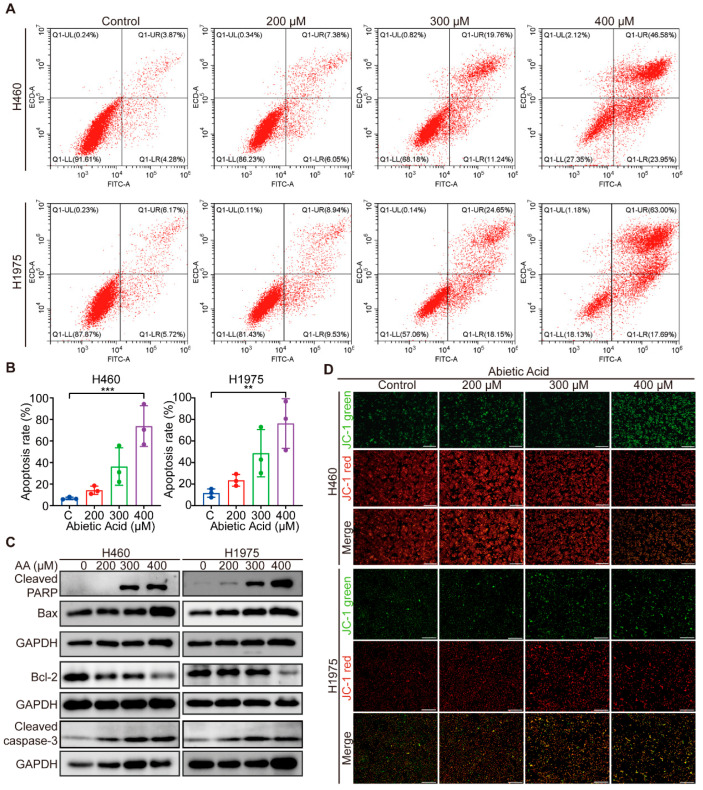
The impact of abietic acid on apoptosis of lung cancer cells. (**A**,**B**) The apoptosis rate of the cells was determined using flow cytometry. Data were shown as the mean ± SD, *n* = 3, ** *p* < 0.01, *** *p* < 0.001. (**C**) The expression of apoptosis-related proteins. Original Western Blot images can be found in Figure S2B. (**D**) The JC-1 fluorescent probe was used to investigate the impact of abietic acid on the mitochondrial membrane potential within lung cancer cells. JC-1 aggregates appear red under fluorescence, while monomers appear green. Scale bar represents 500 μm.

Given the observed cell detachment induced by abietic acid, we next investigated its effect on apoptosis in HELF and lung cancer cells. The results indicated that treatment with 400 μM abietic acid significantly increased the apoptosis rate in both H460 and H1975 cells, with low apoptosis of normal cells, suggesting that abietic acid effectively triggers apoptosis in lung cancer cells (Figure 2A,B and Figure S1). To further validate these findings, the expression of apoptosis-related proteins was analyzed using Western blotting. Intriguingly, high concentrations of abietic acid significantly upregulated the levels of cleaved PARP, Bax, and cleaved caspase-3, while simultaneously downregulating the expression of anti-apoptotic protein Bcl-2 (Figure 2C and Figure S2). Furthermore, following treatment with abietic acid, we observed a gradual reduction in JC-1 accumulation in the mitochondrial matrix, with a shift from red to green fluorescence, as determined using the JC-1 assay kit (Figure 2D). Collectively, these data indicate that abietic acid triggers apoptosis in lung cancer cells, possibly through the mitochondrial pathway.

### 3.3. Network Pharmacology-Based Analysis of the Therapeutic Mechanisms of Abietic Acid in Lung Cancer

**Figure 3:**
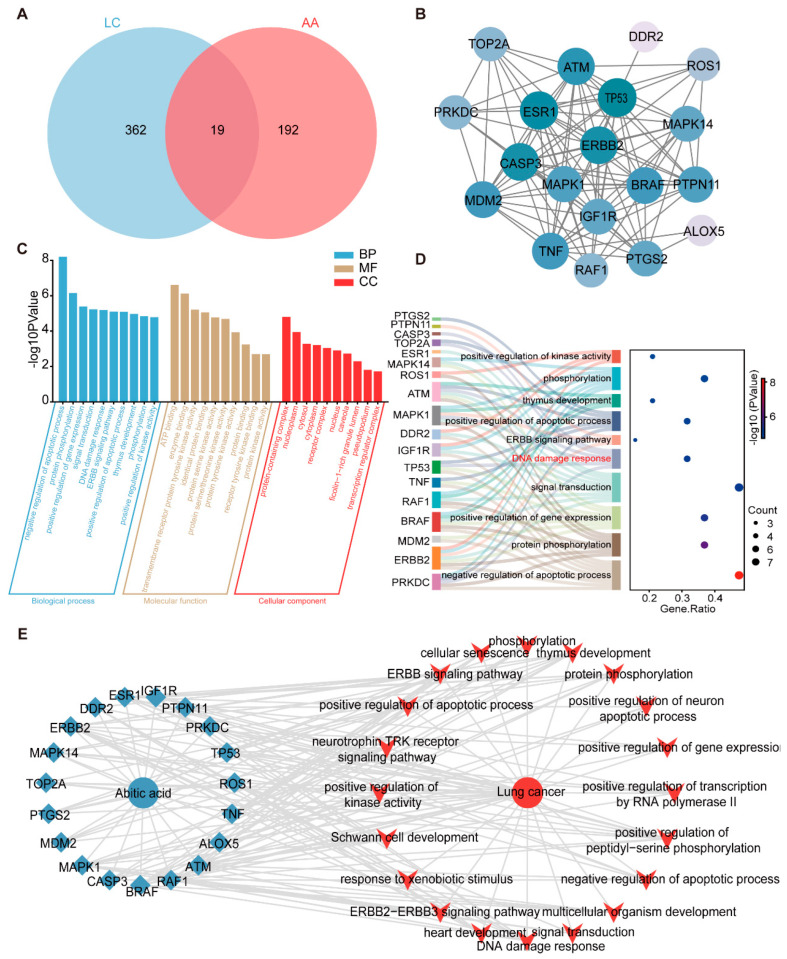
Active targets of abietic acid preventing lung cancer. (**A**) Venn diagrams were used to obtain the unified targets of abietic acid and lung cancer. (**B**) STRING was used to create the PPI network for the potential targets. (**C**) The results of the GO enrichment analysis of 19 targets. (**D**) The Sankey diagram of the top 10 BPs of GO enrichment. (**E**) Drug–target–pathway–disease network. Green dots symbolize compounds, blue rhombuses indicate targets, and red polygons denote pathways.

To further investigate the mechanism by which abietic acid influences lung cancer, we conducted an extensive analysis using network pharmacology. As a result, a total of 211 putative targets of abietic acid and 381 lung cancer-related therapeutic targets were identified, which were retrieved from the aforementioned databases (Supplementary Tables S1 and S2). Subsequently, Venn diagram analysis revealed 19 overlapping targets (Figure 3A), representing candidate proteins potentially involved in the anti-lung cancer activity of abietic acid. In addition, a PPI analysis was conducted on these 19 common targets using the STRING database, and the interaction network was reconstructed and visualized with Cytoscape3.9.1 software. This process yielded a PPI network consisting of 19 nodes and 103 edges (Figure 3B), indicating strong functional associations among these targets. These proteins are considered essential molecular targets through which abietic acid may exert its therapeutic effects in lung cancer.

To further clarify the biological functions of the 19 overlapping targets, a GO enrichment analysis was performed using the DAVID database. As shown in Figure 3C, the top five enriched biological processes (BPs) included the negative regulation of apoptotic process, protein phosphorylation, the positive regulation of gene expression, signal transduction, and DNA damage response. In terms of molecular functions (MFs), the most significant categories were ATP binding, enzyme binding, transmembrane receptor protein tyrosine kinase activity, identical protein binding and protein serine kinase activity. For cellular components (CCs), the top 5 included protein-containing complex, nucleoplasm, cytosol, cytoplasm and receptor complex. The DNA damage response pathway was identified as a core biological process, including six key proteins within the PPI network: TOP2A, PRKDC, CASP3, MAPK1, ATM, and TP53 (Figure 3D). To further explore the interrelationships among the compound, its predicted targets, associated pathways, and disease context, we constructed a “drug–target–pathway–disease” network. This network compromised 41 nodes (19 targets and 20 pathways) and 163 edges and was visualized using Cytoscape (Figure 3E). Taken together, these results suggest that abietic acid may exert its anti-lung cancer activity by regulating multiple targets and signaling pathways, particularly those involved in apoptosis and DNA damage response.

### 3.4. Abietic Acid Induces DNA Damage in Lung Cancer Cells

**Figure 4:**
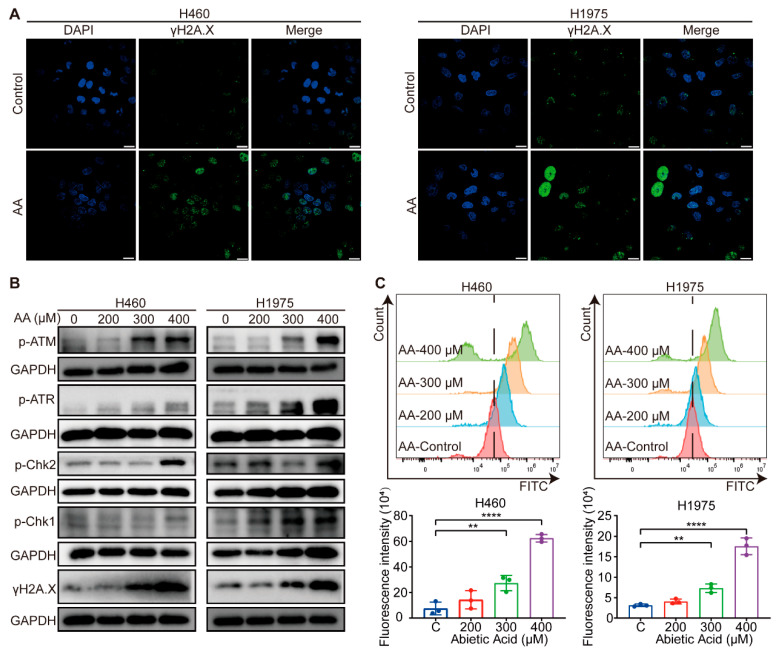
Abietic acid triggered DNA damage in lung cancer cells. (**A**) Representative results of the immunofluorescence assay in H460 and H1975 cells with and without abietic acid treatment. Scale bar represents 10 μm. (**B**) Protein expression of DNA damage-related proteins. Original Western Blot images can be found in Figure S3B. (**C**) The results of ROS levels after the treatment with abietic acid for 48 h. Data are shown as the mean ± SD; *n* = 3; ** *p* < 0.01; **** *p* < 0.0001.

GO enrichment analysis of the 19 potential targets revealed that they are significantly involved in the DNA damage response, a critical cellular process often triggered during cancer therapy [29]. Additionally, γH2A.X is widely recognized as a marker of DNA damage [30]. To validate whether abietic acid induces DNA damage in lung cancer cells, immunofluorescence staining was employed to observe γH2A.X foci in H460 and H1975 cells after treatment with 0 and 300 μM abietic acid for 48 h. As expected, abietic acid treatment caused a notable increase in both the number and fluorescence intensity of γH2A.X foci (Figure 4A), indicating enhanced DNA damage. Furthermore, the expression levels of DNA damage response-related proteins were assessed using Western blotting analysis. These proteins were significantly upregulated in both H460 and H1975 cells in response to abietic acid treatment (Figure 4B and Figure S3). Since oxidative stress is a well-established upstream trigger of DNA damage in cancer cells [31], we also assessed intracellular ROS levels. After exposure to varying concentrations of abietic acid, flow cytometry results showed a significant dose-dependent rise in ROS levels in H460 and H1975 cells (Figure 4C). In summary, these findings provide compelling evidence that abietic acid leads to DNA damage in lung cancer cells.

### 3.5. Abietic Acid Potentially Targets TOP2A to Trigger DNA Damage in Lung Cancer Cells

**Figure 5:**
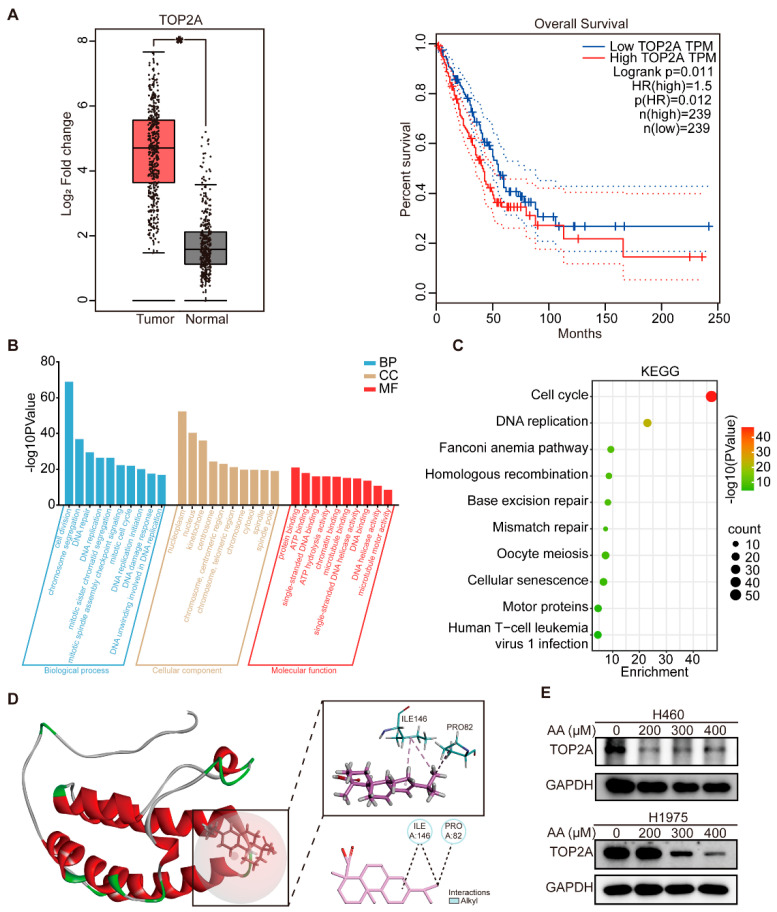
TOP2A was a target of abietic acid, mediating DNA damage in lung cancer. (**A**) The differential expressions of TOP2A in normal and lung cancer tissues and overall survival of patients with differential expressions of TOP2A were obtained from the GEPIA database. * *p* < 0.05. (**B**) The results of GO enrichment analysis of the top 500 gene sets related to TOP2A. (**C**) The results of KEGG enrichment analysis of genes most related to TOP2A. (**D**) Abietic acid exhibits high binding affinity to TOP2A protein via sites of ILE146 and PRO82 through molecular docking analysis. (**E**) The expression of TOP2A in lung cancer after treatment with abietic acid for 48 h. Original Western Blot images can be found in Figure S6B.

Based on the GO enrichment analysis, which identified DNA damage response as a key biological process associated with the 19 predicted targets, six genes (*TOP2A*, *PRKDC*, *CASP3*, *MAPK1*, *ATM*, and *TP53*) were mapped to this pathway (Figure 3D). To further investigate their clinical relevance in lung cancer, we used the GEPIA online tool to analyze the differential expression patterns and patient survival outcomes. Among these genes, only *TOP2A* exhibited significant overexpression in lung cancer tissues relative to normal lung tissues, and high levels of *TOP2A* were significantly linked to lower overall survival in lung cancer patients compared to those with low expression (Figure 5A and Figure S4). Moreover, similar overall survival results were obtained using the Kaplan–Meier plotter (Supplementary Figure S5). These results indicate that TOP2A could be a viable therapeutic target of abietic acid in lung cancer.

To further explore the biological functions related to *TOP2A*, we used Pearson correlation analysis to identify genes strongly correlated with *TOP2A* in the GSE268175 dataset (|R| > 0.5, *p* < 0.05). The resulting gene list is provided in Supplementary Table S3. Subsequently, GO and KEGG pathway analyses were conducted utilizing the 500 most correlated genes. GO biological process analysis revealed that genes associated with *TOP2A* are primarily involved in DNA repair, DNA replication, DNA damage response, and DNA unwinding, which is involved in DNA replication. In terms of cellular components, the enriched terms included the nucleoplasm, nucleus, kinetochore, centrosome and chromosome, and centromeric region. For molecular functions, these genes were strongly associated with single-stranded DNA binding, single-stranded DNA helicase activity, and DNA helicase activity (Figure 5B). In addition, KEGG pathway enrichment analysis highlighted DNA replication and mismatch repair as the primary signaling pathways enriched among *TOP2A*-associated genes (Figure 5C).

These findings collectively demonstrate that TOP2A is essential in DNA damage, prompting the hypothesis that TOP2A serves as a key molecular target through which abietic acid induces DNA damage in lung cancer cells. To explore this possibility, molecular docking was carried out to explore the potential interaction between abietic acid and the TOP2A protein. The PBD number of the receptor protein is TOP2A: 5NNE. Molecular docking results revealed that abietic acid exhibited high binding affinity to the TOP2A protein, with prominent interactions occurring at residues ILE146 and PRO82, and a CDOCKER_Interaction_Energy of −18.8101 kcal/mole (Figure 5D). To experimentally validate the impact of abietic acid on TOP2A expression, H460 and H1975 cells were exposed to varying doses of abietic acid for 48 h. Western blotting analyses revealed a notable decrease in the expression of TOP2A in lung cancer cells (Figure 5E and Figure S6).

**Figure 6:**
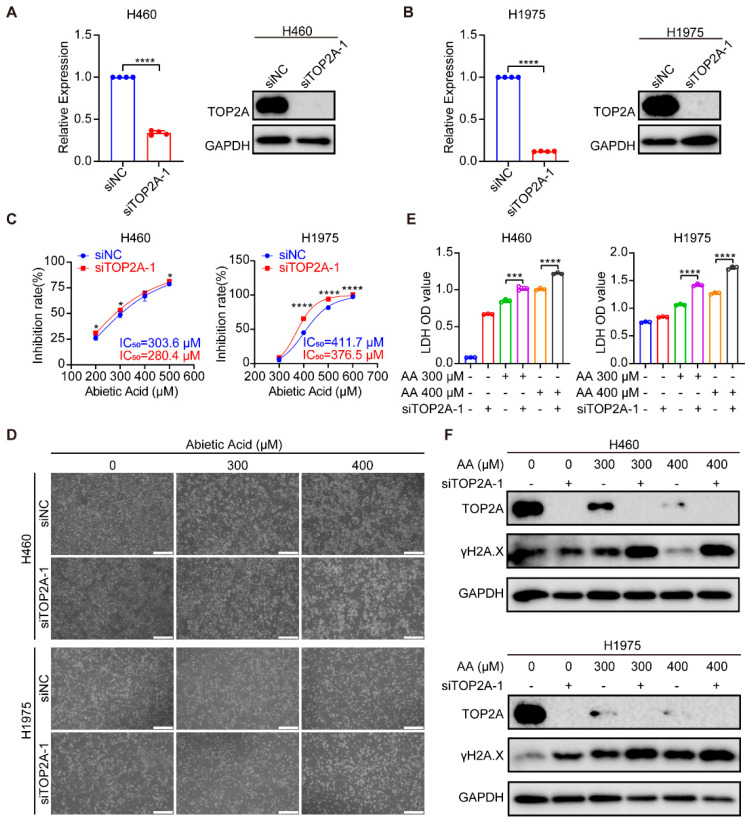
TOP2A knockdown potentiates the anti-growth and DNA-damaging effects of abietic acid in lung cancer cells. (**A**,**B**) Efficiency of TOP2A knockdown in H460 and H1975 cells was validated at the mRNA level by RT-qPCR and at the protein level by Western blotting; mean ± SD, *n* = 4, **** *p* < 0.0001. (**C**) Cell viability assessed by CCK-8 assay. Cells subjected to TOP2A knockdown (siTOP2A-1) or control treatment (siNC) were exposed to the indicated doses of abietic acid for 48 h; mean ± SD, *n* = 3–4, * *p* < 0.05, **** *p* < 0.0001. (**D**) Representative micrographs showing morphological changes in H460 and H1975 cells treated with abietic acid for 48 h, with or without TOP2A knockdown. Scale bar: 500 μm. (**E**) Cytotoxicity measured by LDH release assay in cells treated with abietic acid for 48 h, with or without TOP2A knockdown; mean ± SD, *n* = 3, *** *p* < 0.001, **** *p* < 0.0001. (**F**) Western blot analysis of TOP2A and the DNA damage marker γH2A.X protein expression in cells treated with abietic acid for 48 h, with or without TOP2A knockdown. Original Western Blot images can be found in Figure S7C,D.

To further elucidate the role of the abietic acid-induced downregulation of TOP2A activation in mediating cell growth inhibition and DNA damage in lung cancer cells, TOP2A knockdown experiments were performed. RT-qPCR and Western blotting analysis confirmed TOP2A knockdown in mRNA and protein expression (Figure 6A,B and Figure S7A). Subsequently, the knockdown of TOP2A in H460 and H1975 cells significantly increased cell inhibition after abietic acid treatment for 48 h (Figure 6C). Importantly, microscopic observations and LDH release assays revealed that TOP2A knockdown in H460 and H1975 cells attenuated cellular proliferation and induced cell death (Figure 6D,E). Furthermore, TOP2A knockdown intensified cell DNA damage, showing a significant increase in the expression of γH2A.X compared to cells treated with abietic acid alone (Figure 6F and Figure S7B). Similar results were also observed following the knockdown of TOP2A using siTOP2A-2 (Supplementary Figure S8). These results provide both computational and experimental evidence that TOP2A is a target of abietic acid, mediating its DNA damage effects in lung cancer cells.

### 3.6. Abietic Acid Possessed Anticancer Activity in Vivo

**Figure 7:**
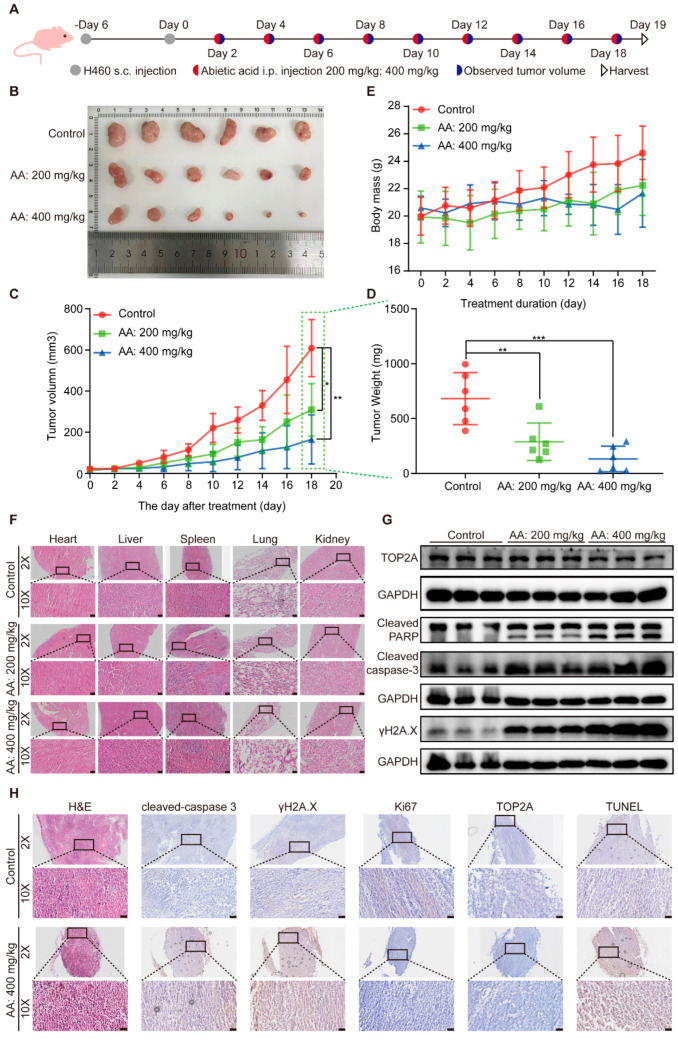
The effect of abietic acid on BALB/c nude mice with H460 tumor. (**A**) The experimental design included subcutaneous (s.c.) tumor inoculation and treatment protocol in H460 tumor-bearing athymic nude mice. Abietic acid was given at doses of 200 mg/kg and 400 mg/kg every other day, starting six days post-tumor implantation. (**B**) Images of resected tumors from mice. (**C**) The average body weight of mice with H460 tumors during treatment. Data are shown as the mean ± SD; *n* = 6; * *p* < 0.05; ** *p* < 0.01. (**D**) The volumes of tumors were recorded. Data are shown as the mean ± SD; *n* = 6; ** *p* < 0.00; *** *p* < 0.001. (**E**) The weights of tumors were recorded. (**F**) H&E staining was used to analyze sections of the heart, liver, spleen, lung, and kidney in a lung cancer mouse model. Scale bar represents 50 μm. (**G**) The expression of TOP2A, cleaved PARP, cleaved caspase 3 and γH2A.X proteins. Scale bar represents 50 μm. Original Western Blot images can be found in Figure S10B. (**H**) H&E and IHC staining of Cleaved caspase-3, γH2A.X, Ki67, TOP2A, and TUNEL of tumor section. Scale bar corresponds to 50 μm.

To further validate the anticancer properties of abietic acid on lung cancer in vivo, a subcutaneous tumor-bearing nude mouse model of lung cancer was established using H460 cells. We set up abietic acid administration groups with different doses of 200 and 400 mg/kg and subjected them to intraperitoneal injections every other day for a duration of 19 days (Figure 7A). The results indicated that abietic acid significantly decreased tumor weight and volume compared to the control group (Figure 7B–D). Additionally, the body weights of the nude mice remained stable throughout the experimental period (Figure 7E), indicating good systemic tolerance. Histological analysis using H&E staining, along with blood biochemical analyses, obtained no detectable toxicity in important organs like the heart, liver, spleen, lungs, and kidneys (Figure 7F and Figure S9), supporting the low toxicity profile of abietic acid. These results demonstrate that abietic acid effectively inhibited lung cancer tumor growth in vivo without causing noticeable toxic side effects in nude mice.

Furthermore, Western blotting analysis of tumor tissues confirmed an upregulation of cleaved caspase-3, cleaved PARP, and γH2A.X proteins, accompanied by a downregulation of TOP2A protein expression in the treated groups relative to the control group (Figure 7G and Figure S10). Additionally, H&E staining and immunohistochemistry of tumor tissues revealed disrupted tumor cell morphology, along with elevating the expression of cleaved caspase-3, γH2A.X, and TUNEL-positive cells, and decreasing the expression of TOP2A and the proliferation marker Ki67 in the treatment groups (Figure 7H). These findings, observed in vivo, align with the results obtained in vitro and collectively demonstrate that abietic acid exerts significant antitumor effects in lung cancer by inducing DNA damage and apoptosis without causing substantial systemic toxicity.

## 4. Discussion

Lung cancer is a prevalent malignant tumor globally, with increasing incidence and mortality rates, particularly due to factors such as air pollution and smoking [32]. At present, adjuvant chemotherapy or radiotherapy following surgery is the mainstay of lung cancer treatment. However, achieving further efficacy proves challenging due to the significant side effects and limited tolerability associated with chemoradiotherapy [33]. Adjuvant therapy using TCM with standard treatments for NSCLC has been shown to prolong progression-free survival and reduce mortality, while potentially decreasing the recurrence and metastasis of tumors following surgery [34,35]. The role of TCM in controlling symptoms associated with NSCLC treatment is increasingly recognized as vital. Chinese herbal medicines exhibit the potential to treat complex diseases through the synergistic effects of multiple components, diverse targets, and several channel mechanisms [36]. Network pharmacology is a research methodology that integrates large-scale bioinformatics data and drug databases [37]. This method represents a paradigm shift from the traditional ‘single drug, single target, single disease’ model, instead adopting a comprehensive research strategy that investigates drug therapeutic mechanisms through the lens of multiple bioactive components, diverse molecular targets, and interconnected biological pathways [38].

In recent decades, natural products remain a rich and proven source of bioactive compounds for medicinal chemistry research and drug discovery [39]. These natural compounds demonstrate various biological effects, including anti-inflammatory, anti-microbial, and, notably, anti-tumor activities properties. Abietic acid is a biologically active compound sourced from traditional herbal medicine *Pimenta racemosa* var. *grissea*. In this study, we investigated the therapeutic potential of abietic acid against lung cancer. We discovered from cytology experiments that abietic acid exerts strong growth-inhibitory effects on lung cancer cells. In addition, we observed that abietic acid triggered apoptosis via disrupting the mitochondrial membrane potential in lung cancer cells. Apoptosis is a programmed cell death modality, which is divided into two pathways: intrinsic cascade and extrinsic cascade [40]. The identification of natural products capable of inducing apoptosis in cancer cells has a significant impact on the advancement of strategies to inhibit cancer growth and progression [41]. When apoptosis occurs, cell morphology changes. The process of apoptosis includes cell membrane coiling and blistering, a reduction in cell volume, nucleus condensation, chromatin condensation and the formation of apoptotic bodies [42]. In mitochondrial-mediated apoptosis, the mitochondrial membrane potential drops, leading to the release of cytochrome C into the cytoplasm, where it interacts with Apaf-1 and activates caspase-9, and this active cytochrome C/Apaf-1/caspase-9 complex activates caspase-3 and caspase-7, leading to apoptosis [43,44].

In this study, we performed an extensive screening of the CHEMBL, NetInfer, and SwissTargetPrediction databases to identify potential targets for abietic acid. This analysis led to the identification of 211 candidate targets. Additionally, 381 lung cancer-associated targets were obtained from Disgenet online databases. By comparing these two sets, we discovered 19 overlapping targets using a Venn diagram. According to the GO analysis, these 19 predicted targets were mainly involved in DNA damage response. Furthermore, to confirm the findings of network pharmacology, immunofluorescence and Western blotting analyses were performed to confirm that abietic acid induces DNA damage in lung cancer cells. After lung cancer were exposed to abietic acid, we observed an increase in the fluorescence intensity of γH2A.X, a well-established marker of DNA damage. Moreover, abietic acid significantly increased the phosphorylation levels of ATR, ATM, Chk1, Chk2, and H2A.X. More importantly, abietic acid exposure substantially augmented intracellular ROS in lung cancer, which may contribute to the observed DNA damage.

Moreover, the GEPIA analysis results indicated that TOP2A was the only gene with markedly elevated expression in lung cancer tissues relative to normal tissues. Notably, higher levels of TOP2A expression levels were significantly associated with poorer overall survival. To gain more insight into the biological functions related to TOP2A, we performed functional enrichment analyses. The results demonstrated that the top 500 genes related to TOP2A were strongly linked to the DNA damage pathway. From these results, we hypothesize that TOP2A may serve as a key target for DNA damage induced by abietic acid in lung cancer. The following protein analysis using Western blotting indicated that abietic acid inhibited the expression of TOP2A in lung cancer cells. In addition, abietic acid showed high binding affinities to the TOP2A protein. The enzyme TOP2A is essential for overcoming topological issues during DNA replication, transcription, and repair, and has been recognized as a target for cancer treatment [45]. Studies have shown that when DNA damage occurs in cancer cells, nuclear transglutaminase 2 can rapidly accumulate at DNA double-strand break sites, and directly interact with TOP2A to promote DNA double-strand break repair, which will lead to drug resistance in cancer cells [46,47,48]. Several clinically active agents targeting TOP2A, such as etoposide, doxorubicin, and mitoxantrone, have shown promise as a therapy for solid tumors and hematologic cancers [49,50]. Therefore, exploring natural TOP2A inhibitors from Chinese herbal medicine could provide valuable opportunities for cancer chemoprevention and therapy.

One potential limitation of this study is that the in vivo doses required to achieve significant pharmacological effects were higher than those of some typical clinical medications. We recognize that these higher doses may raise concerns about their clinical applicability. However, the primary objective of this study was to elucidate the efficacy and mechanisms of abietic acid in lung cancer. At this exploratory stage, using relatively high doses helps maximize the detection of its biological effects in complex systems and assess its maximal therapeutic potential. In addition, research has reported that abietic acid has low cytotoxicity to normal cells [21,51]. Moreover, some clinically successful drugs require high-dose preclinical treatment in the early stages of development. Metformin, for example, which is a first-line treatment for type 2 diabetes, often demonstrates efficacy in preclinical in vivo studies at doses of 225–250 mg/kg in mice [52,53]. Future studies will aim to optimize dosing strategies by developing targeted delivery systems, such as nanoparticles, liposomes, or antibody–drug conjugates, or by exploring combination therapy regimens to reduce the dose of a single drug, thereby enhancing clinical translatability and safety while maintaining efficacy [54,55,56,57].

While our data strongly suggest that TOP2A is a crucial target for abietic acid, we must consider the possibility of off-target effects, which are common for many natural compounds. In particular, the observed cytotoxic effects and DNA damage response (evidenced by γH2A.X elevation) may not be solely attributable to direct TOP2A inhibition and could be partly mediated through ROS-dependent pathways. It is important to note that TOP2A inhibition and ROS generation are not mutually exclusive. As TOP2A is a key enzyme in maintaining genomic stability, its dysfunction can lead to the accumulation of DNA double-strand breaks and replication collapse, thereby triggering replication stress and increasing intracellular ROS levels [58,59,60,61]. Therefore, the increased ROS signal detected in this study may indeed be a downstream event of TOP2A inhibition. These events could collectively form a self-amplifying signaling loop, ultimately driving the cell toward death. Nonetheless, we cannot rule out the possibility that abietic acid directly induces ROS generation, independently of the TOP2A pathway. Delineating the precise contribution of direct TOP2A targeting versus primary ROS induction to abietic acid’s cytotoxicity remains an important objective for future studies.

Our research results indicate that abietic acid induces DNA damage in lung cancer cells by downregulating TOP2A. Doxorubicin, etoposide, and teniposide are clinical TOP2A inhibitors used to treat lung cancer. These classic TOP2 poisons exert their effects by stabilizing the TOP2-DNA cleavage complex [62]. This effectively traps the enzyme in the DNA, converting transient breaks in the DNA into permanent, lethal double-strand breaks [49,63]. Although these drugs are highly effective, they can cause side effects, such as genotoxicity and bone marrow suppression. Notably, doxorubicin can cause cumulative cardiotoxicity [64,65]. Furthermore, the extensive DNA damage caused by these drugs can increase the risk of secondary malignancies [62]. By contrast, our data suggest that abietic acid primarily reduces TOP2A protein levels rather than directly poisoning the enzyme–DNA complex. This suggests that abietic acid may have different safety characteristics to conventional TOP2 inhibitors, potentially alleviating the severe genotoxicity associated with them. As a naturally derived terpene compound with a novel mechanism of action, abietic acid is a promising drug candidate for further development to overcome the limitations of existing TOP2A-targeted therapies.

This study demonstrated the high potential of abietic acid, a compound that targets TOP2A, in the discovery of drugs for treating lung cancer. It is expected to be used as a therapeutic agent against lung cancer in the future. However, there are still some short-comings of this study: food consumption was not quantitatively measured in vivo, and the stability of abietic acid binding to TOP2A, the pharmacokinetics, and the clinical safety of abietic acid need further study.

## 5. Conclusions

**Figure 8:**
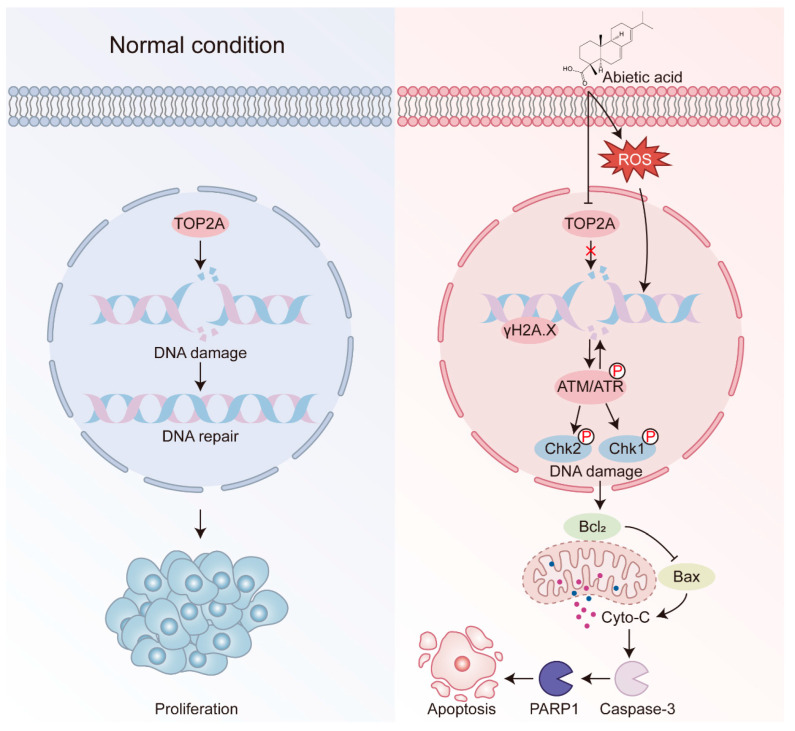
Schematic diagram for the functional mechanism of the abietic acid anti-tumor. On entering the lung cancer cells, abietic acid downregulates TOP2A to induce DNA damage, and further induces apoptosis through the intrinsic mitochondrial apoptotic pathway.

In conclusion, our study highlights the anticancer potential of abietic acid in lung cancer (Figure 8). Abietic acid was found to effectively suppress the growth of lung cancer cells, trigger cell apoptosis, and cause DNA damage. Network pharmacology analyses revealed TOP2A as a key molecular target, a finding further supported by molecular docking and Western blotting validation. An in vivo study demonstrated significant tumor suppression following abietic acid administration in xenograft mouse models without causing notable systemic toxicity, as evidenced by their stable body weights, normal histological features of major organs, and unaltered blood biochemical parameters. These findings highlight abietic acid as a promising, low-toxicity natural compound with potential for further development as a therapeutic agent for lung cancer.

## Data Availability

Raw data for the original contributions presented in this study are included in the Supplementary Materials. Further inquiries can be directed to the corresponding author.

## Supplementary Materials

The following supporting information can be downloaded at: https://www.mdpi.com/article/10.3390/biom15111498/s1, Figure S1: Flow cytometric quantification of apoptosis in HELF cells treated with abietic acid; Figure S2: Relative protein expression levels of apoptosis-related proteins; Figure S3: Relative protein expression levels of DNA damage-related proteins; Figure S4: Bioinformatic analysis of candidate genes in lung cancer from GEPIA; Figure S5: Kaplan–Meier survival analysis of lung cancer patients based on TOP2A expression across independent cohorts. Figure S6: Relative protein expression levels of TOP2A; Figure S7: Relative protein expression levels after transfection with siTOP2A-1; Figure S8: The anti-growth effects of abietic acid following siTOP2A-2 transfection; Figure S9: The key serum biochemical parameters in mice; Figure S10: Relative protein expression levels in mice; Table S1: 211 active targets of abietic acid; Table S2: 381 lung cancer-related therapeutic targets; Table S3: Pearson correlation analysis with TOP2A in GSE268175.
