# Supplementary material for: Abietic Acid Induces DNA Damage and Cell Apoptosis in Lung Cancer Cells Through Targeting TOP2A
Source: Biomolecules. 2025 Oct 24;15(11):1498. doi: 10.3390/biom15111498 (PMC12650274; doi:10.3390/biom15111498)
Supplement: Supplementary file 1 [file biomolecules-15-01498-s001.zip › Supplementary materials.pdf]

Abiectic acid induces DNA damage and cell apoptosis in lung cancer cells through targeting TOP2A

Zhiyu Zhu[1, 2], Jie Gu[1], Zehua Liao[1], Mengting Chen[1], Yun Wang[1], Jingyi Song[1], Jing Xia[1], Xinbing Sui[1], Shuang Lin[3, *], Xueni Sun[1, *]

[1] School of Pharmacy, Hangzhou Normal University, Hangzhou, Zhejiang 311121, China

[2] College of Life and Environmental Sciences, Hangzhou Normal University, Hangzhou, Zhejiang 311121, China

[3] Department of Thoracic Surgery, the First Affiliated Hospital, College of Medicine, Zhejiang University, Hangzhou, Zhejiang 310058, China

*Corresponding authors to: xnsun@hznu.edu.cn (X.S.), shuanglin@zju.edu.cn (S.L.).

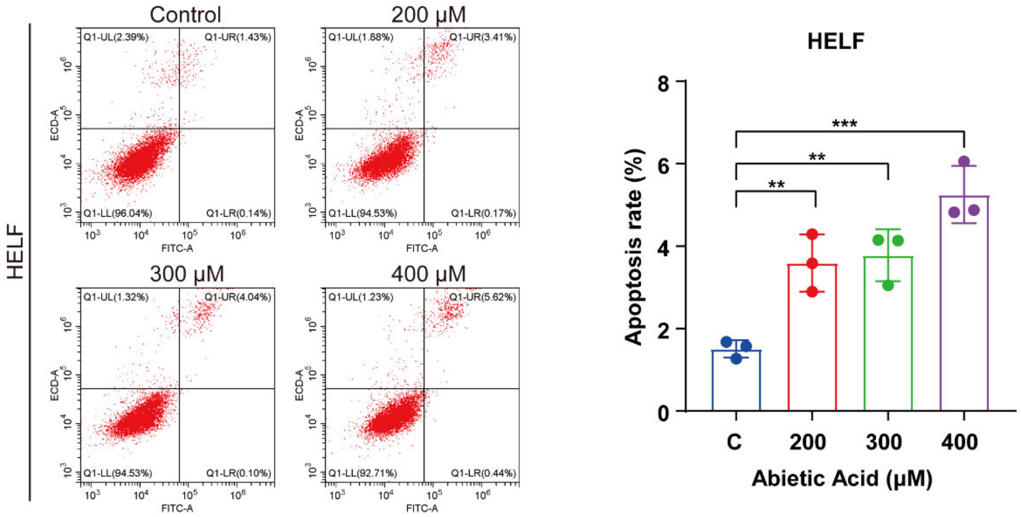

Figure S1. Flow cytometric quantification of apoptosis in HELF cells treated with abietic acid. Data were shown as the mean ± SD, n=3, ** P<0.01, *** P<0.001.

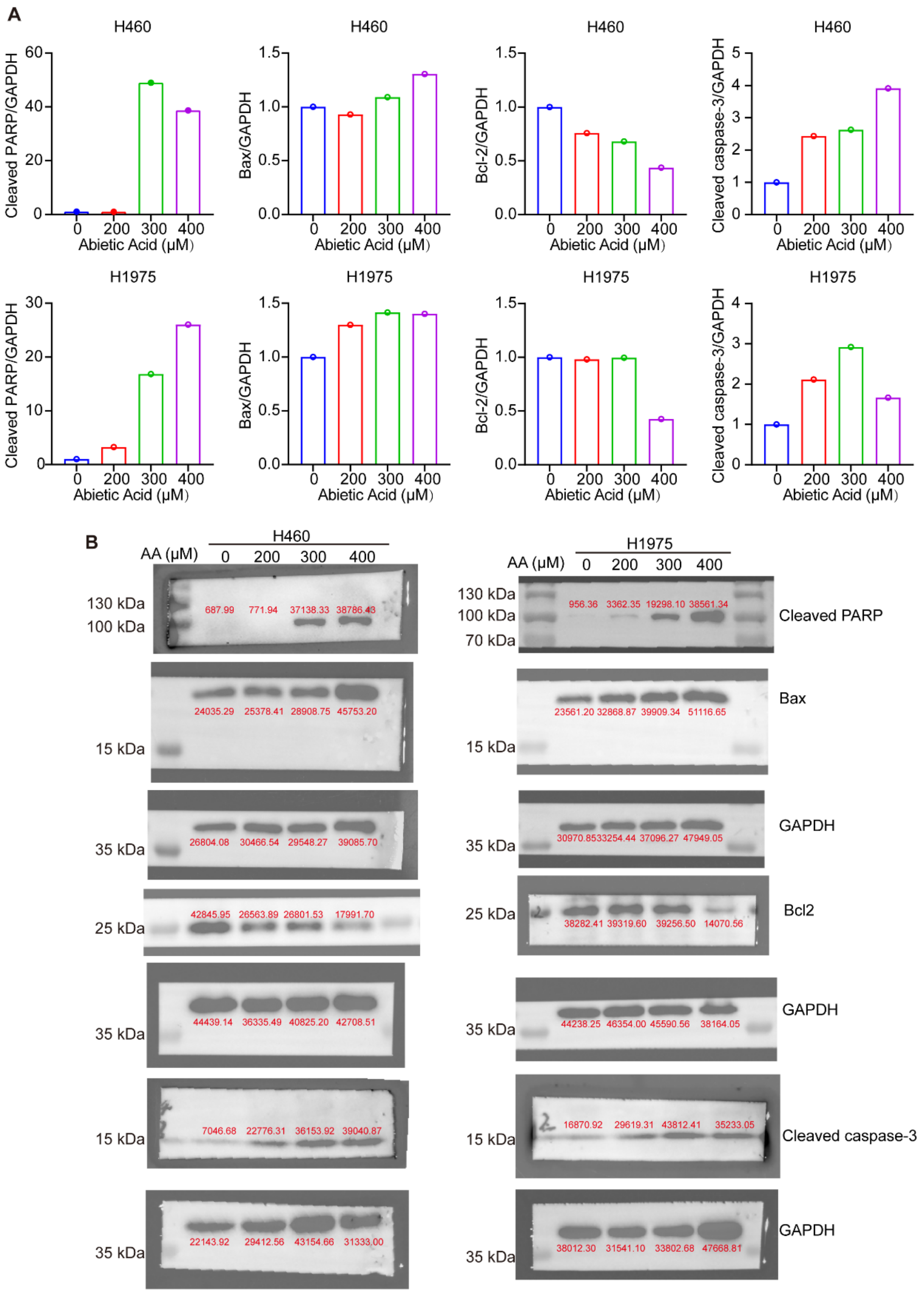

Figure S2. (A) Relative protein expression levels of cleaved PARP, Bax, Bcl-2, and cleaved caspase-3 following 48 h treatment with abietic acid, as quantified by densitometric analysis of Western blots and normalized to GAPDH. **(B)** Original Western blot membranes corresponding to the data presented in panel (A). The grayscale values are annotated in red on the respective bands.

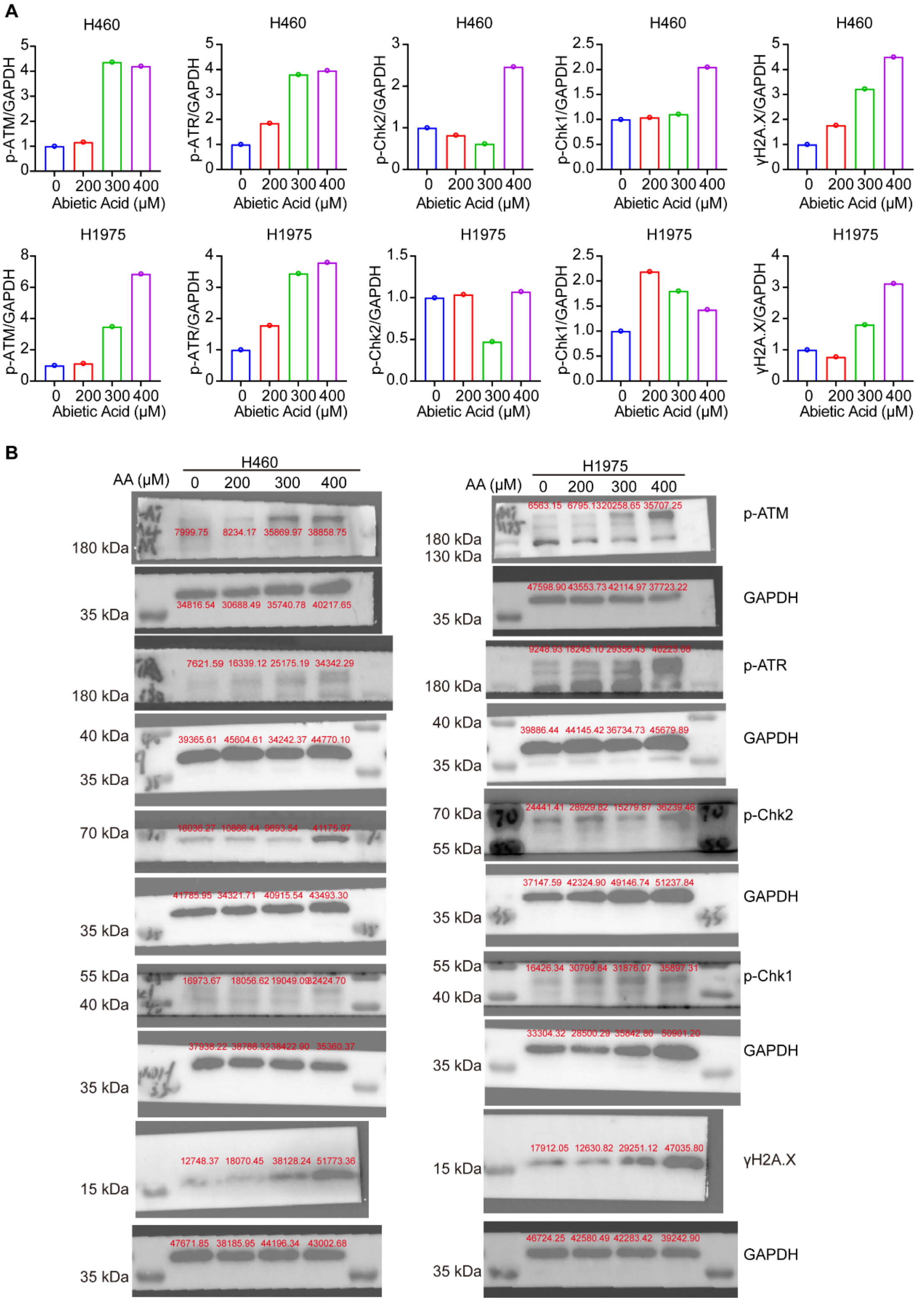

Figure S3. (A) Relative protein expression levels of p-ATM, p-ATR, p-Chk2, p-Chk1, and γH2A.X following 48 h treatment with abietic acid, as quantified by densitometric analysis of Western blots and normalized to GAPDH. **(B)** Original Western blot membranes corresponding to the data presented in panel (A). The grayscale values are annotated in red on the respective bands.

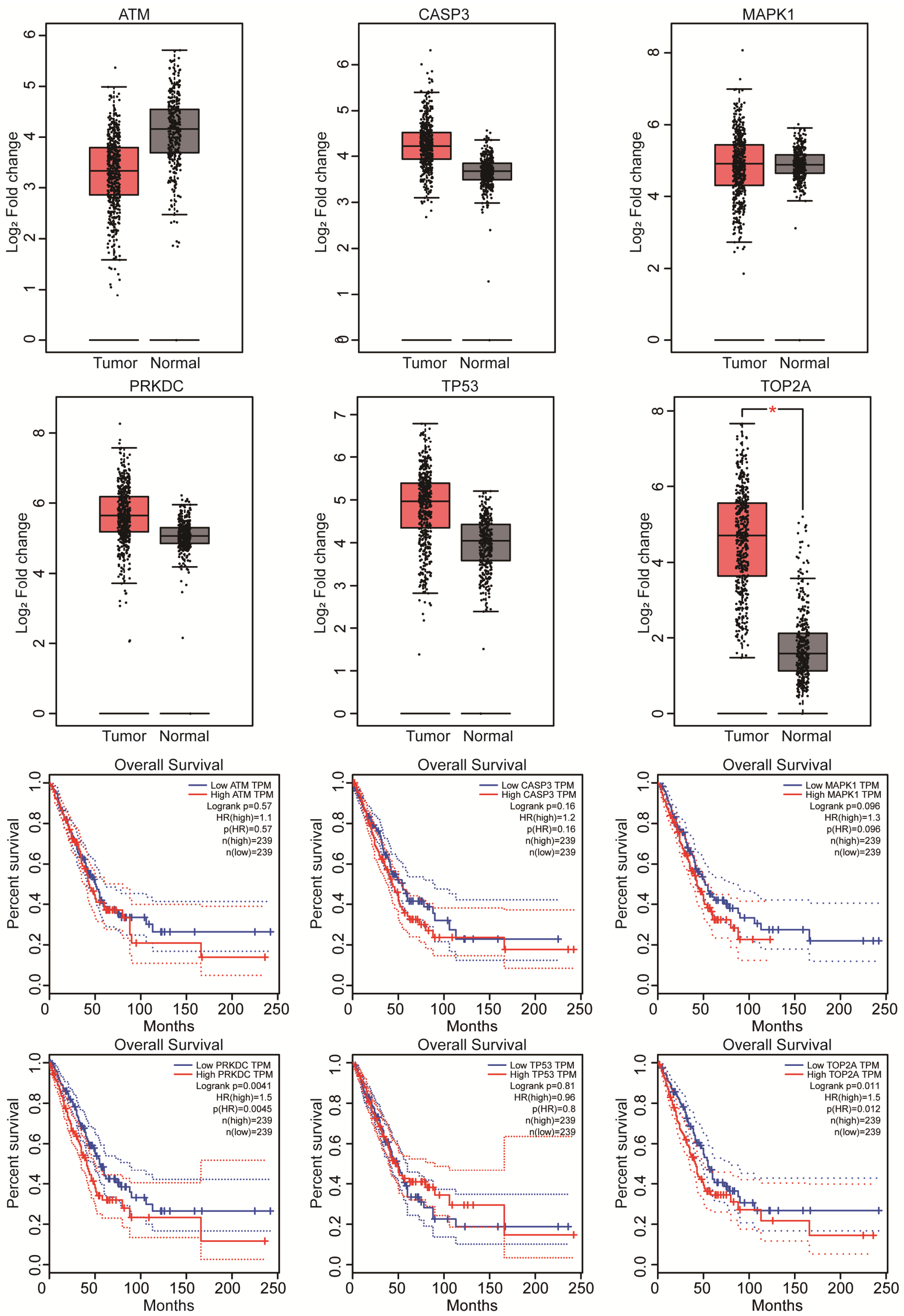

Figure S4. Bioinformatic analysis of candidate genes in lung cancer from GEPIA. Box plots illustrate the transcriptomic expression of *ATM, CASP3, MAPK1, PRKDC, TP53,* and *TOP2A* across normal and primary lung cancer tissues, and Kaplan-Meier curves depict the overall survival of lung cancer patients based on high (red) and low (blue) expression levels of each gene.

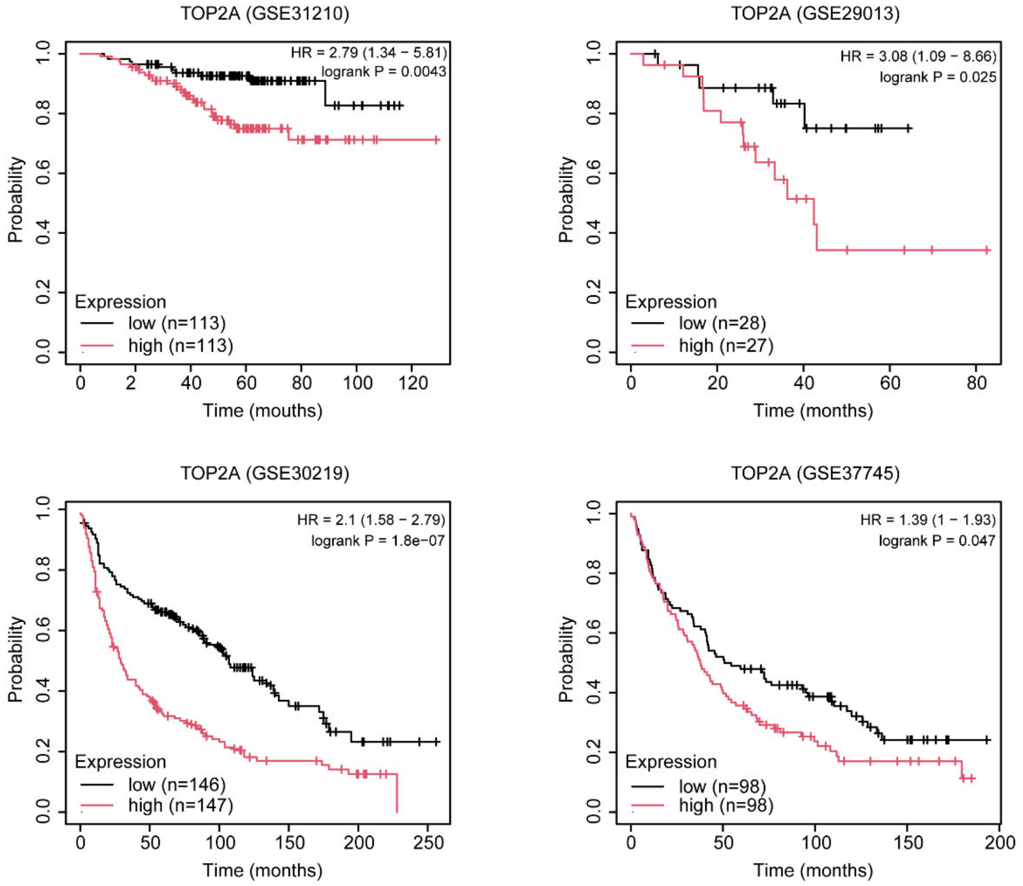

Figure S5. Kaplan-Meier survival analysis of lung cancer patients based on TOP2A expression across independent cohorts. Overall survival was analyzed using the Kaplan-Meier plotter for patients with high (red) and low (blue) TOP2A expression levels in four independent datasets: GSE31210, GSE29013, GSE30219, and GSE37745.

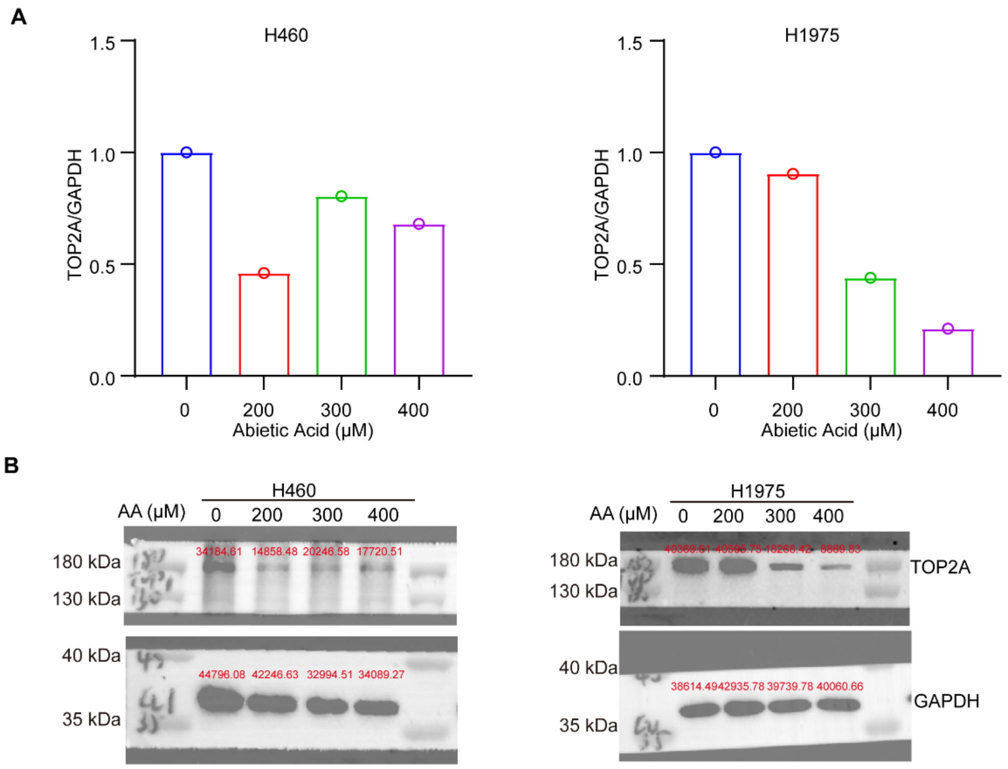

Figure S6. (A) Relative protein expression levels of TOP2A following 48 h treatment with abietic acid, as quantified by densitometric analysis of Western blots and normalized to GAPDH. **(B)** Original Western blot membranes corresponding to the data presented in panel (A). The grayscale values are annotated in red on the respective bands.

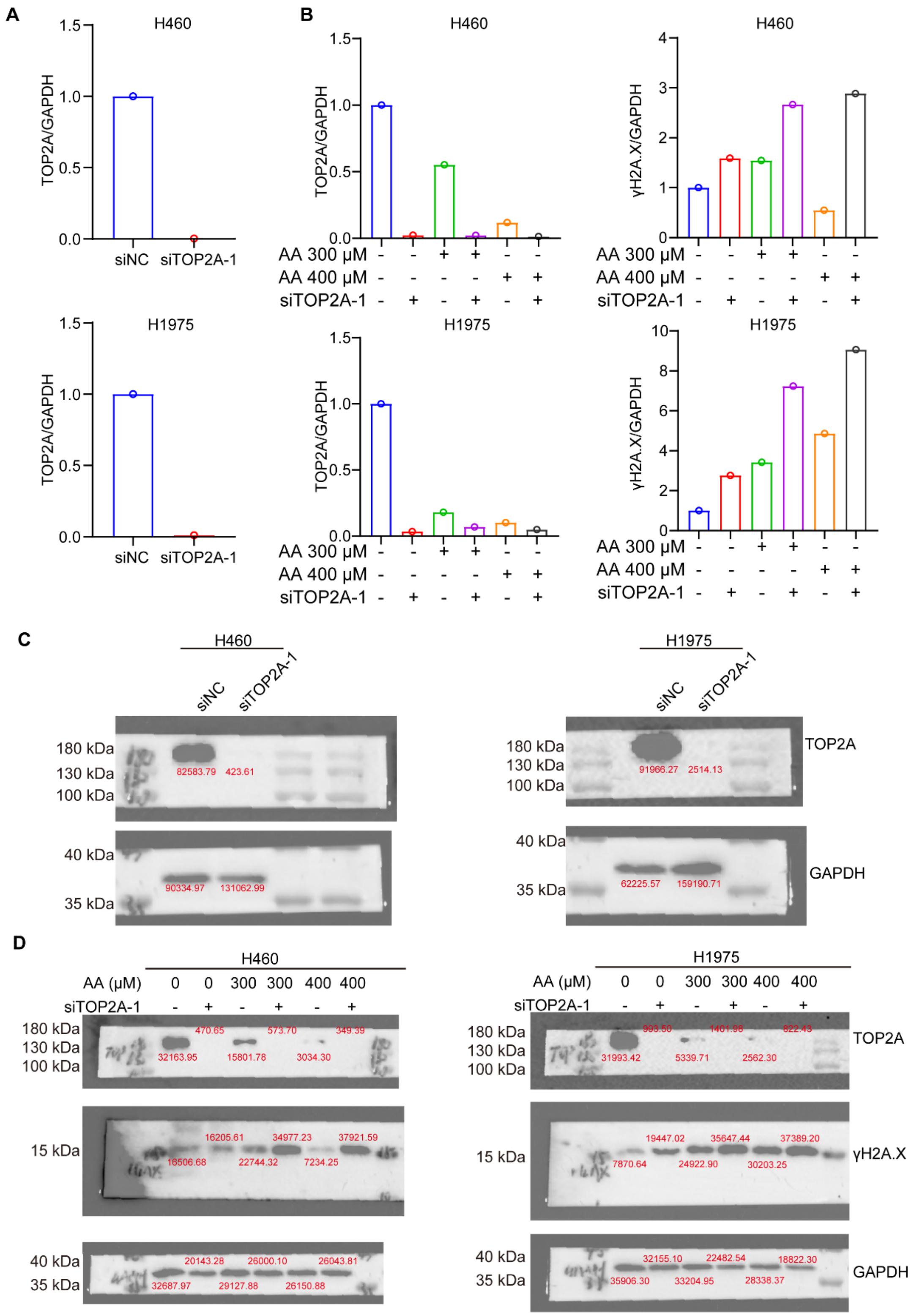

Figure S7. (A) Relative TOP2A protein levels in H460 and H1975 cells after transfection with TOP2A-targeting siRNA (siTOP2A-1), confirming knockdown efficiency. **(B)** Relative protein expression of TOP2A and the DNA damage marker γH2A.X in H460 and H1975 cells treated with abietic acid for 48 h, with or without prior TOP2A knockdown. **(C-D)** Original Western blot membranes corresponding to the data

presented in panels (A) and (B), respectively. The grayscale values used for densitometric analysis are annotated in red.

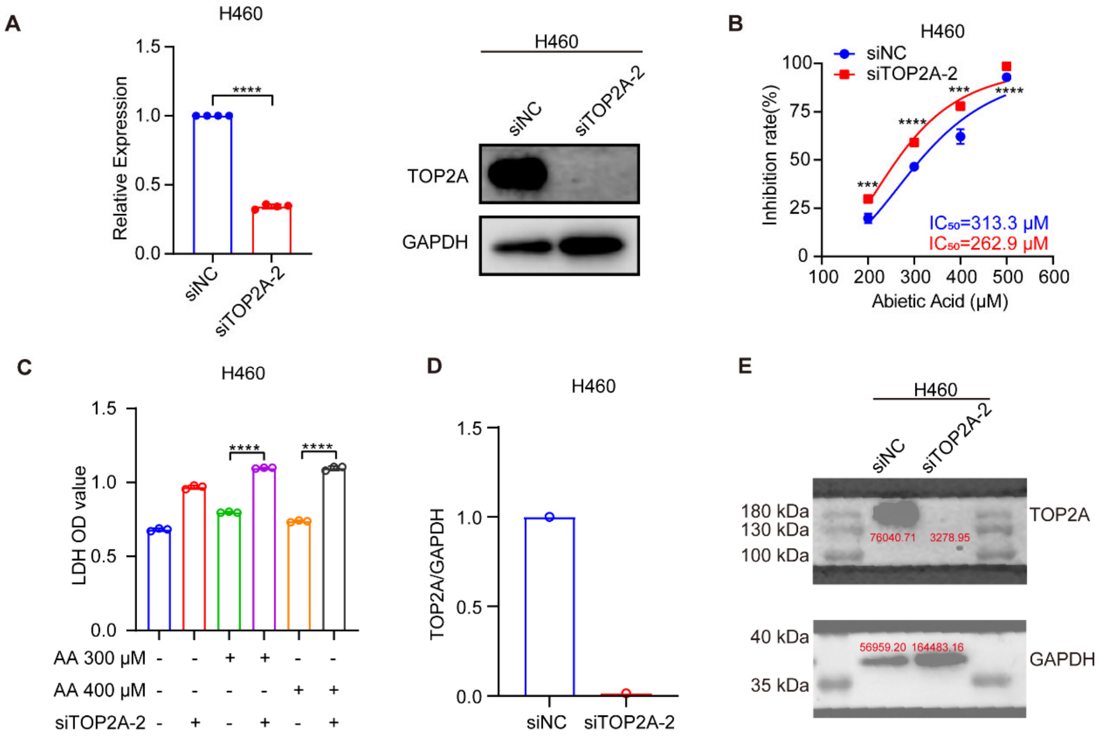

Figure S8. (A) Efficiency of TOP2A knockdown in H460 cell was validated at the mRNA level by RT-qPCR and at the protein level by Western blotting; mean ± SD, n=4, **** P<0.0001. **(B)** Cell viability assessed by CCK-8 assay. Cells subjected to TOP2A knockdown (siTOP2A-2) or control treatment (siNC) were exposed to the indicated doses of abietic acid for 48 h; mean ± SD, n=4, *** *P*<0.001, **** *P*<0.0001. **(C)** Cytotoxicity measured by LDH release assay in cells treated with abietic acid for 48 h, with or without TOP2A knockdown; mean ± SD, n=3, **** *P*<0.0001. **(D)** Relative TOP2A protein levels in H460 cell after transfection with TOP2A-targeting siRNA (siTOP2A-2), confirming knockdown efficiency. **(E)** Original Western blot membranes corresponding to the data presented in panel (D). The grayscale values used for densitometric analysis are annotated in red.

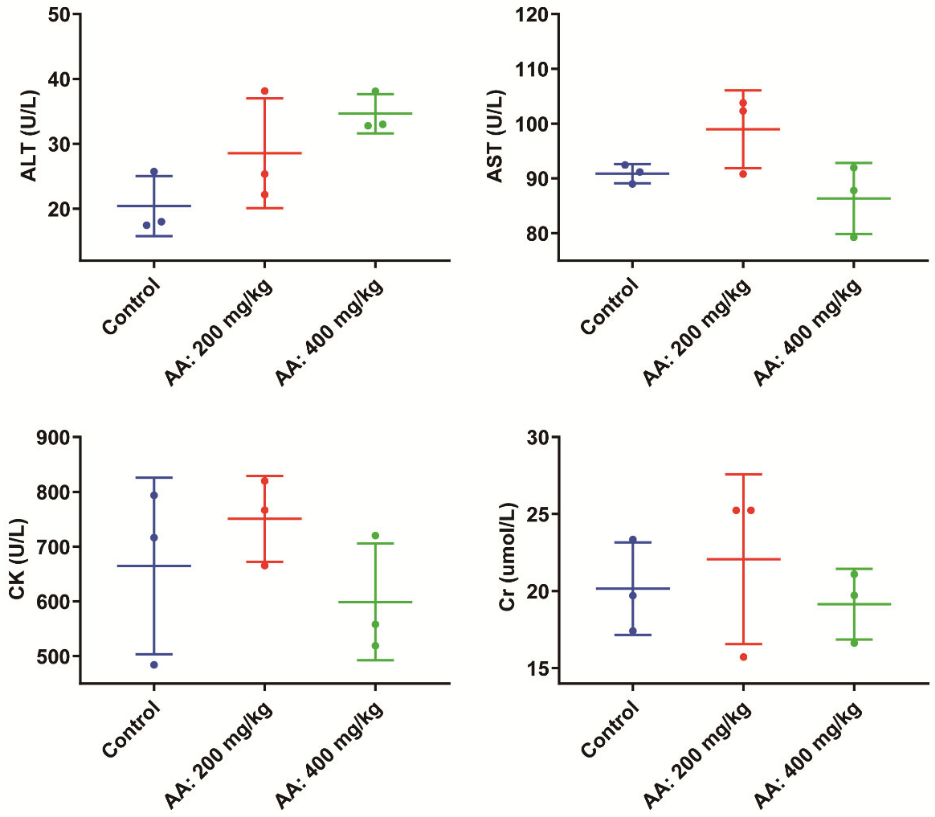

Figure S9. Analysis of key serum biochemical parameters in mice. The concentrations of alanine aminotransferase (ALT), aspartate aminotransferase (AST), creatine kinase (CK), and creatinine (Cr) in mouse serum were quantified.

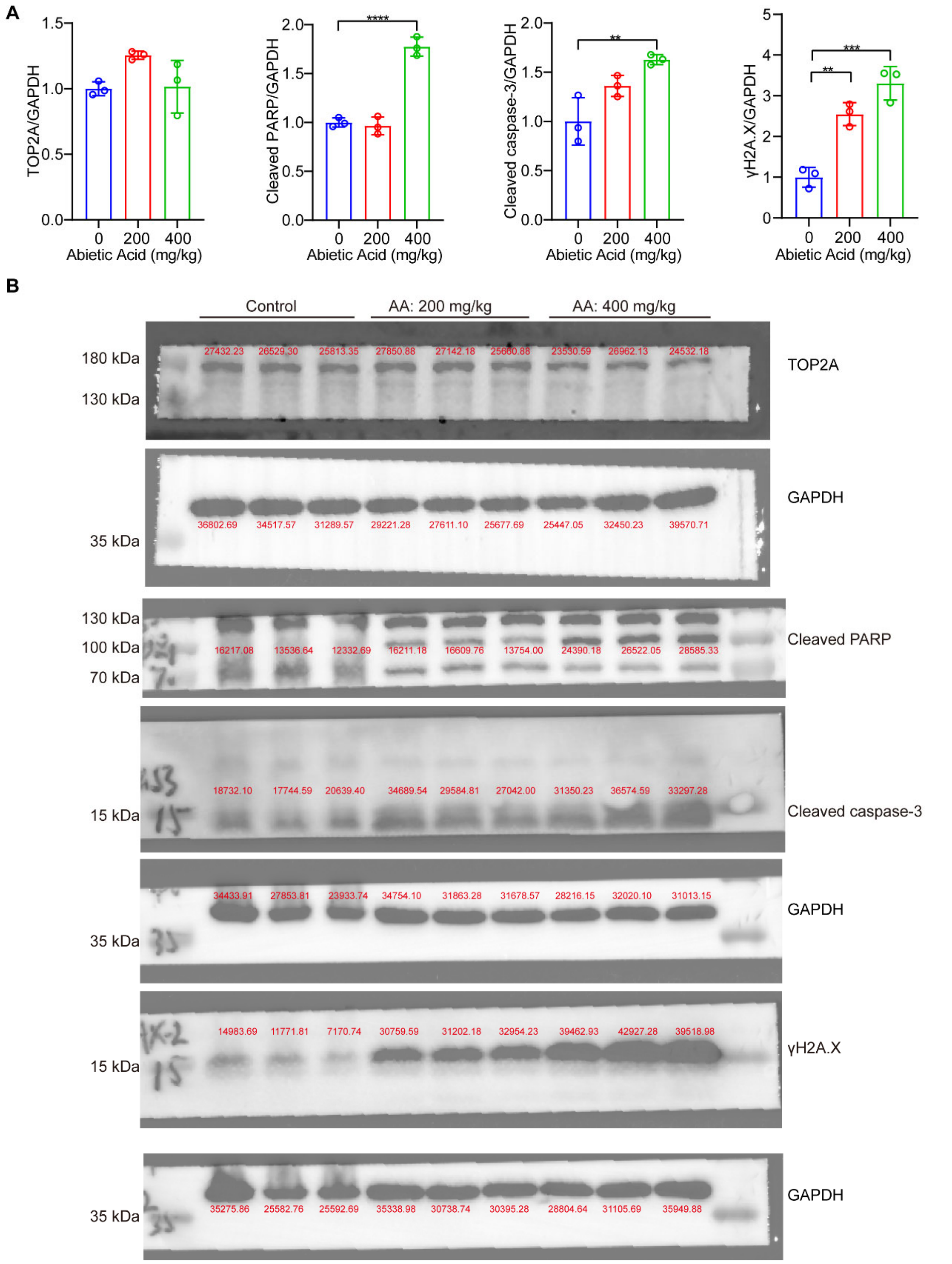

Figure S10. (A) Relative protein expression levels of TOP2A, cleaved PARP, cleaved caspase-3, and γH2A.X proteins in mice treated with abietic acid; mean ± SD, * *P*<0.05, ** *P*<0.01, *** *P*<0.001, **** *P*<0.0001. **(B)** Original Western blot membranes corresponding to the data presented in panel (A). The grayscale values are annotated in red on the respective bands.
